# The Bushland, Texas, maize evapotranspiration, growth, and yield dataset Collection

**DOI:** 10.1038/s41597-025-04539-2

**Published:** 2025-02-04

**Authors:** Steven R. Evett, Gary W. Marek, Paul D. Colaizzi, Karen S. Copeland, Brice B. Ruthardt, Terry A. Howell

**Affiliations:** USDA Agricultural Research Service, Conservation & Production Research Laboratory, Bushland, Texas USA

**Keywords:** Hydrology, Climate-change impacts

## Abstract

A collection of datasets describing six years of experiments on maize (*Zea mays*, L.) (corn) is presented (1989, 1990, 1994, 2013, 2016, and 2018). Four weighing lysimeters were used to determine crop evapotranspiration (ET). In-soil and above ground microclimate and ET data are presented on a 15-minute interval as are weather data for all days of the year. Data analysis determined ET, precipitation, irrigation, and dew and frost accumulation on a 15-minute basis from lysimeter mass data. Soil water content data from calibrated neutron probe readings is presented on a periodic basis. Crop planting, harvest, fertilization, pest control, and other agronomic information are presented in agronomic calendars by day of year. Crop growth data are presented on a periodic basis throughout the growing season, as are final crop biomass and yield data. The data are suitable for analysis of effects of irrigation and other agronomic decisions on crop yield and water productivity in the Southern High Plains region of the USA, for model calibration and testing, and for model improvement.

## Background & Summary

**Fig. 1:**
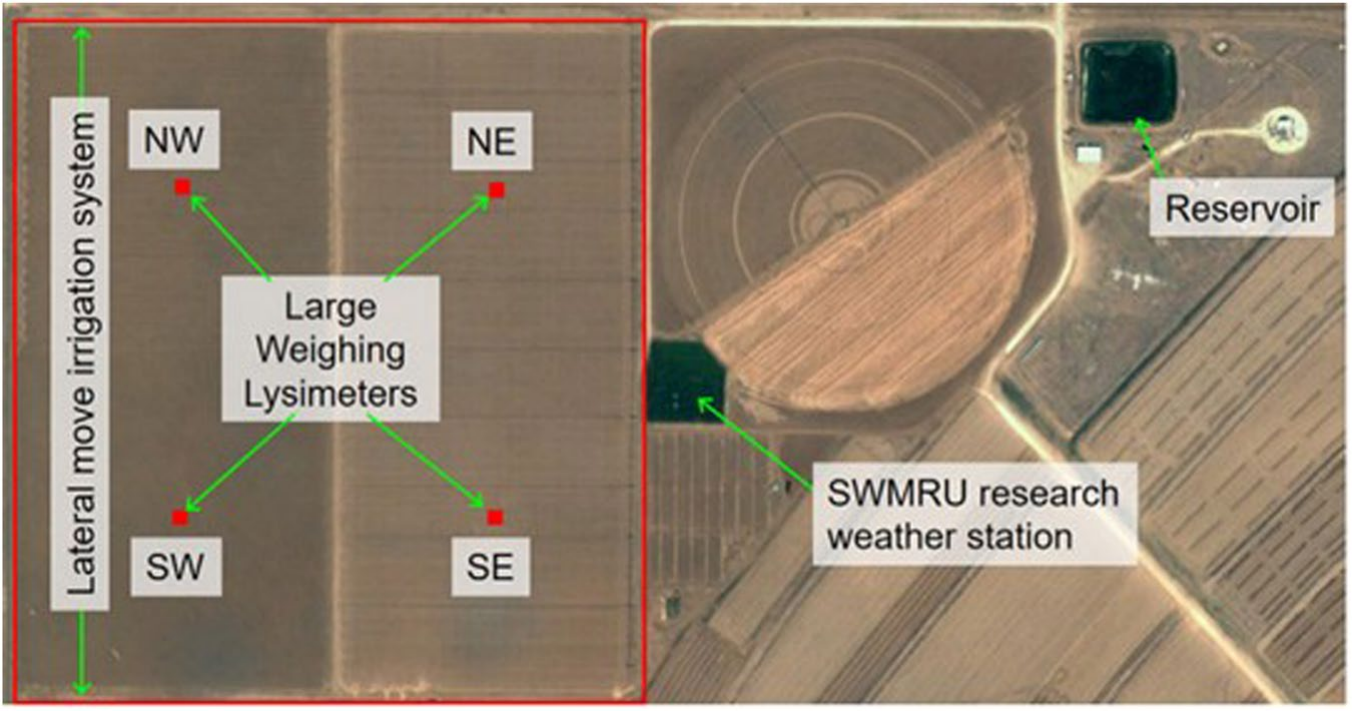
USDA ARS Bushland, Texas, irrigation, weighing lysimeter, weather, and related facilities in 2012. The NE and SE fields were irrigated using subsurface drip irrigation beginning in 2013. Before 2013, all fields were irrigated with the lateral move sprinkler irrigation system when not in dryland production. The four fields are arranged in a square, outlined in red in the illustration.

In 1987 and 1988, four large, precision weighing lysimeters^1^ were built by USDA ARS at Bushland, Texas for the purpose of measuring by mass balance the evapotranspiration (ET) of the major crops grown on the southern High Plains as influenced by crop choice, variety, agronomic practices (tillage, applications of fertilizers and pesticides, etc.) (Fig. 1), irrigation management, and irrigation application method^2^. Initial purposes included providing regionally specific crop coefficients^3–5^ for irrigation management that would be used in a regional scheduling network, developing and testing alternative methods of ET determination, and developing quality-controlled datasets for crop model testing, improvement, and intercomparison^6–9^. For the latter purpose, not only ET data but soil water content^10^ and temperature, energy and water flux, plant growth and yield, and weather^11^ data were collected, and methods for quality control of all data streams were developed^12,13^. This report describes the organization and details of a collection of datasets^14^ for each growing season (year) when maize (*Zea mays*, L., also known as corn in the United States) was grown for grain at the USDA-ARS Conservation and Production Laboratory (CPRL), Soil and Water Management Research Unit (SWMRU), Bushland, Texas USA (Lat. 35.186714°, Long. −102.094189°, elevation 1170 m above MSL).

## Methods

In any given cropping season, maize was grown on between two and four large, precision weighing lysimeters, each in the center of a 4.44 ha square field. The four fields were contiguous and arranged in four quadrants, which were labeled northeast (NE), southeast (SE), northwest (NW), and southwest (SW) (Fig. 1). See the resource titled “Geographic Coordinates, USDA, ARS, Bushland, Texas” for UTM geographic coordinates of field and lysimeter locations. The land slope is <1% and topography is flat. The mean annual precipitation is ~460 mm (1991–2021 mean), the 20-year pan evaporation record indicates ~2,600 mm Class A pan evaporation per year, and winds are typically from the South and Southwest. The climate is semi-arid with ~70% (320 mm) of the annual precipitation occurring from May to September, during which period the pan evaporation averages ~1520 mm.

Maize was grown on only the NE and SE fields in 1989 and 1990, and on all four fields in 1994, 2013, 2016, and 2018. Irrigation was by linear move sprinkler system in 1989, 1990, and 1994, and that system was equipped with various application technologies such as high-pressure impact sprinklers, low pressure spray applicators, and low energy precision applicators (LEPA). In 2013, 2016, and 2018, two lysimeters and their respective fields (NE and SE) were irrigated using subsurface drip irrigation (SDI)^15,16^, and two lysimeters and their respective fields (NW and SW) were irrigated by a linear move sprinkler system equipped with spray applicators. Daily logs were kept of all field operations (tillage, fertilizer and pesticide applications, planting, plant measurements, harvest, etc.), lysimeter maintenance and hand tillage, and manual measurements that could impact lysimeter readings. These logs are in the dataset, “Agronomic Calendars for the Bushland, Texas Maize for Grain Datasets”^17^.

### Soil water sensing and irrigation management

Irrigations designated as “full” were managed to replenish soil water used by the crop on a weekly or more frequent basis as determined by soil profile water content readings. Irrigations designated as less than full (i.e. Deficit) were applied as a percentage of the full irrigation amount. Readings were made with a field calibrated neutron probe from 0.10- to 2.40-m depth in 0.20-m depth increments in the field. Readings in the two access tubes in each lysimeter were made at depths from 0.10- to 1.90-m, again at 0.20-m depth increments. The number and spacing of neutron probe reading field locations changed through the years (additional sites were added), which is one reason why subsidiary datasets and data dictionaries are needed to specify those locations. Resulting soil water content data are in the dataset, “Soil Water Content Data for The Bushland, Texas Large Weighing Lysimeter Experiments”^10^. In the 2013 and later years, soil water sensing using electromagnetic sensors was done in the lysimeters as described in the section on weighing lysimeters. Fields were planted by machine (six-row planter) at pre-determined densities, and lysimeters and areas near the lysimeters that could not be machine planted were hand planted to a greater seeding density. After crop emergence, replicated field plant stand counts determined the resulting machine planted field plant density, and plants on the lysimeters and surrounding hand-planted areas were hand thinned to match the field plant density. The lysimeters and fields were planted to the same row spacing (0.76 m) and depth. Tillage depth (by hand on the lysimeters and by machine in the fields), and fertilizer and pesticide applications were also the same between fields and lysimeters.

### Weighing lysimeter measurement and instrumentation

The weighing lysimeters were calibrated using masses traceable to NIST^18^ and used to measure mass, which was converted to relative soil water storage in mm depth of water considering the density of water and the surface area of the lysimeter. Calibration confirmed accuracy of 0.05 mm or better for data acquired at 5-minute intervals (mean of 300 readings taken on 6 s intervals). The 5-minute change in soil water storage (∆*S*, mm) was used along with precipitation (*P*, mm), dew and frost accumulation (DW, mm), irrigation amount (*I*, mm), the sum of runon and runoff (*R*, mm), and soil water flux into the lysimeter soil volume (*F*, mm) to calculate crop evapotranspiration (ET) using the soil water balance equation.

1 $${\rm{ET}}=\triangle S+P+{\rm{DW}}+I+F+R$$

Fields were commonly furrow diked to reduce runon and runoff and the monolith container freeboard was approximately 50 mm so that *R was* negligible except during infrequent very large precipitation events. In the rare event that *R* was not negligible, data were flagged to indicate that ET data were unreliable. Because the lysimeter soil monolith was contained in a steel box, *F* was zero unless drainage occurred in which case it was negative. The lysimeters were equipped with vacuum drainage systems operating at 1-m water head of suction, and drainage was captured in tanks suspended from the lysimeter monolith such that drainage into the tanks did not change the mass of the lysimeter. Drainage under the conditions of these studies was small enough that tanks sometimes did not fill during a year. When tanks did fill they were emptied and the change in mass recorded by the lysimeter weighing system was converted to an equivalent depth of water, resulting in a negative *F* value during the period of tank emptying, which typically was 15 minutes or less.

In addition to the measurable components of the soil water balance shown in Eq. 1, a weighing lysimeter system can include virtual components (Eq. 2). These include any additions to or subtractions from the lysimeter mass that are not due to water balance components. For example, scale counterweight adjustments (CW) made to keep the scale load cell within its operational range cause changes in relative water storage not related to ET. Two of the four lysimeters are equipped with subsurface drip irrigation (SDI) systems that utilize water storage tanks suspended from the lysimeter so that they are weighed by the lysimeter scale. Irrigation of the lysimeters commences with filling of the tanks, which causes a step change in the lysimeter apparent relative water storage, which itself is easily quantified when identified. As the irrigation proceeds the tanks are emptied, but the apparent relative water storage is not affected. The step change from tank filling constitutes a water storage gain, which is designated as I in Eq. 1. As part of regular yearly maintenance, however, the SDI lines must be flushed and for that the tanks must be filled and then emptied as water is pumped through the drip lines to flush them of accumulated sand and debris. Because the soil water content is negligibly affected, the filling and emptying of irrigation tanks for purposes of drip line flushing are virtual components of the water balance designated by FL and DT, respectively. Occasionally, other lysimeter mass changes not related to ET occur due to operations and maintenance and must be accounted for. These are indicated by a “V” flag. Including the virtual components, the water balance equation becomes.

2 $${\rm{ET}}=\triangle {\rm{S}}+{\rm{P}}+{\rm{DW}}+{\rm{I}}+{\rm{F}}+{\rm{R}}+{\rm{CW}}+{\rm{FL}}+{\rm{DT}}+{\rm{V}}$$

Amounts of ET, change in storage, irrigation, precipitation and dew and frost accumulation were determined from changes in relative water storage using a spreadsheet developed for this purpose^19,20^.

Each lysimeter was equipped with a suite of instruments to sense precipitation, wind speed, air temperature and humidity, radiant energy (incoming and reflected, typically both shortwave and longwave), surface temperature, soil heat flux, and soil temperature, all of which are reported at 15-minute intervals. In some years, radiant energy at the soil surface was sensed, as well as photosynthetically active radiation (PAR). Precipitation was sensed at the lysimeters using tipping bucket rain gauges at fixed-height masts, and data from them are questionable when crop height was greater than the gauge orifice height. For this and other reasons discussed in the section on Weather data, precipitation data from gauges at the lysimeters are considered only indicative of the occurrence of precipitation and sprinkler irrigation, not definitive of amounts of these. In 2013 and later years, sensors (model TDR315L, Acclima, Inc., Meridian, ID, USA) were installed at depths of 0.02 and 0.06 m above soil heat flux plates to determine soil water content, temperature, and bulk electrical conductivity so that soil heat flux sensed at 0.08-m depth could be corrected to the surface. Instruments used changed from season to season, which is another reason that subsidiary datasets and data dictionaries for each season are required. Ensemble, this dataset is called “Weighing Lysimeter Data for The Bushland, Texas Maize for Grain Datasets”^21^. The data dictionaries describe the instrument used to sense each environmental parameter in each year. Important conventions concerning the data-time correspondence, sign conventions, and terminology specific to the USDA ARS, Bushland, TX, field operations are given in the resource titled “Conventions for Bushland, TX, Weighing Lysimeter Datasets”.

A separate dataset was developed using more exacting and time-consuming processing of 5-minute lysimeter data and called the “Evapotranspiration, Irrigation, Dew/frost - Water Balance Data for The Bushland, Texas Maize for Grain Datasets”^22^. This more carefully quality controlled dataset contains 15-minute and daily (midnight to midnight) mean values of evapotranspiration, irrigation, precipitation, dew and frost accumulation, and drainage tank emptying data. These data are considered the Bushland data of record for ET, irrigation, precipitation, and dew/frost accumulation amounts that would be used for simulation model calibration, testing, improvement, and intercomparison.

### Plant growth and yield measurement

As mentioned previously, plant stand was measured during and through the completion of emergence to determine the density of plants in the machine-planted fields. Measurements were made in multiple replicate plots in each field, and the number of replicates and sizes of plots did change from year to year and so are recorded in the data dictionaries for the datasets named “Growth and Yield Data for the Bushland, Texas Maize for Grain Datasets”^23^. On an approximately biweekly basis, as determined by weather and other circumstances that prevented entrance to the fields, growth stage, plant height, plant row width, leaf area index, and above ground biomass were measured in replicate plots in each of the four fields, while plant height and plant row width only were measured on the four weighing lysimeters. Leaf area index and above-ground biomass were determined from destructive harvest of all above-ground biomass, which was separated into leaves and stems, weighed, then dried at 60 °C until constant mass, and then weighed again. Before drying, the leaves were run through a digital scanning bed leaf area meter (model LI-3100, LI-COR, Lincoln, Neb. USA), which was frequently calibrated using test disks, to determine total leaf area.

Both hand harvest in replicate plots in each field and machine harvest (combine) in replicate measured areas in each field were accomplished after crop maturity. For hand harvest, the entire above-ground biomass from each replicate plot in the field and from each lysimeter was harvested and separated into cobs, leaves, and stems, each of which were weighed then dried at 60°C until constant mass, then weighed again. Cobs were shelled and grain number, total grain mass, and mass per grain were measured. Harvest index was reported on a dry mass basis, and yields are reported as both dry grain Mg/ha and as bushels per acre at standard moisture content.

### Weather data

In addition to meteorological data sensed at each lysimeter, weather data were gathered from replicate calibrated sensors at 2-m and 10-m heights over grass mowed to 0.12-m height on a 0.4-ha flat weather station adjacent to the NE and SE lysimeter fields (Fig. 1). The weather station grass was irrigated by flood from 1989 to 1993 and with subsurface drip irrigation from 1994 to present so as to maintain a reference grass surface (well-watered and fertilized, cut to standard height). Precipitation was measured with a tipping bucket rain gauge with 20-cm orifice. Solar irradiance was sensed with Epply and Licor solar irradiance sensors. Wind speed and direction were sensed with wind speed and direction sensors at both 2-m and 10-m heights. Relative humidity and air temperature sensors^24^ were mounted in both a cotton belt shelter and at 2-m height on the mast. Reported precipitation data were determined from weighing lysimeter mass increase during the event, converted to an equivalent depth of water. Precipitation data from the tipping bucket gauges at the weather station and at the lysimeters were used as a check on precipitation data from the weighing lysimeter mass changes, but weighing lysimeter data were reported because the weighing lysimeters have a much larger effective orifice (~9 m^2^ compared with ~0.03 m^2^ for the rain gauge), and the lysimeter effective orifice is practically at ground surface so that wind has negligible effect on catch, unlike the case for the rain gauges at the weather station and weighing lysimeters. The weighing lysimeters typically report larger amounts of precipitation from large, quick storms than do the rain gauges, and they also typically are sensitive to small precipitation events that are not recorded by the tipping bucket rain gauges on the research station. Because convective thunderstorms common to the region have rain shafts that deliver precipitation to relatively small areas, precipitation can vary spatially. Therefore, precipitation is reported separately for each of the four lysimeters when available and a mean value is reported as well. Models and manufacturers of weather station sensors changed over the years as documented in the Excel spreadsheets for each year. Calibrated sensors were always used and in most cases sensors were replicated to provide data for quality control and gap filling^2^. Quality control and data gap filling were practiced as reported by Evett *et al*.^12^, and data are reported on a 15-minute basis for each year in the datasets in “Standard Quality Controlled Research Weather Data – USDA-ARS, Bushland, Texas”^11^.

## Data Records

All data records are stored on the USDA ARS National Agricultural Library Ag Data Commons (https://agdatacommons.nal.usda.gov/) in the dataset collection named, “The Bushland, Texas Maize for Grain Datasets” (10.15482/USDA.ADC/1526317)^14^. There are six datasets, each containing multiple files:

1. Agronomic Calendars for the Bushland, Texas Maize for Grain Datasets^17^
2. Growth and Yield Data for the Bushland, Texas Maize for Grain Datasets^23^
3. Weighing Lysimeter Data for The Bushland, Texas Maize for Grain Datasets^21^
4. Evapotranspiration, Irrigation, Dew/frost - Water Balance Data for The Bushland, Texas Maize for Grain Datasets^22^
5. Standard Quality Controlled Research Weather Data – USDA-ARS, Bushland, Texas^11^
6. Soil Water Content Data for The Bushland, Texas Large Weighing Lysimeter Experiments^10^

Each dataset and the files in it are described in the following.

Agronomic Calendars for the Bushland, Texas Maize for Grain Datasets^17^.

For each year there is a crop calendar for the two east lysimeters (NE and SE), if maize was grown there, and another calendar for the two west lysimeters (NW and SW), if maize was grown there. The agronomic calendar for each season lists by date the agronomic operations on the Bushland, TX, large weighing lysimeters and surrounding fields, including tillage, planting, fertilization, pesticide application, furrow diking, irrigations, etc., and also sensor installation, sensor reading that might disturb lysimeter operation (neutron probe readings), maintenance operations such as emptying drainage tanks, adjusting lysimeter scale counterweights, electronic and electrical maintenance, etc. Amounts and kinds of fertilizer and pesticide applications are given with proper chemical names and SI units. Calendars are in Excel files consisting of two tabs, the first a data dictionary, and the second containing the calendar.

Resources in this dataset:

- Resource Title: 1989 Bushland, TX, east maize agronomic calendar. File Name: 1989_East_Maize_Calendar.xlsx.
- Resource Title: 1990 Bushland, TX, east maize agronomic calendar. File Name: 1990_East_Maize_Calendar.xlsx.
- Resource Title: 1994 Bushland, TX, east maize agronomic calendar. File Name: 1994_East_Maize_Calendar.xlsx.
- Resource Title: 1994 Bushland, TX, west maize agronomic calendar. File Name: 1994_West_Maize_Calendar.xlsx.
- Resource Title: 2013 Bushland, TX, east maize agronomic calendar. File Name: 2013_East_Maize-Calendar.xlsx.
- Resource Title: 2013 Bushland, TX, west maize agronomic calendar. File Name: 2013_West_Maize_Calendar.xlsx.
- Resource Title: 2018 Bushland, TX, west maize agronomic calendar. File Name: 2018_West_Maize_Calendar.xlsx.
- Resource Title: 2018 Bushland, TX, east maize agronomic calendar. File Name: 2018_East_Maize_Calendar.xlsx.
- Resource Title: 2016 Bushland, TX, west maize agronomic calendar. File Name: 2016_West_Maize_Calendar.xlsx.
- Resource Title: 2016 Bushland, TX, east maize agronomic calendar. File Name: 2016_East_Maize_Calendar.xlsx.

Growth and Yield Data for the Bushland, Texas Maize for Grain Datasets (10.15482/USDA.ADC/1526331)^23^.

Growth and yield data are contained in Excel files containing several tabs that list by date, field, and replicate number: crop growth [plant height, crop row width, leaf area index, biomass (undried and dried), ear mass (when present), growth stage, etc.]; population density; machine (combine) yield by field location; and hand (manual) harvest data per replicated plot (number of plants and ears, total biomass, dry grain yield, yield at standard moisture content, harvest index). There are two growth and yield files for each year, one for east (NE and SE) fields if maize was grown there, and one for west (NW and SW) fields if maize was grown there. Protocols for plant measurements and growth staging changed over the years, requiring different data dictionaries for difference growing seasons. Protocols are explained in data dictionary tabs that precede each data tab. Data dictionaries explain the header for each column of data in the like-named data tab. Explanations include units of measure, plot size or row length sampled, and any pertinent methodological information. Data are from replicate (n = 3 or greater) samples in the field and non-destructive (except for final harvest) measurements on the weighing lysimeters. In most cases yield data are available from both manual sampling on replicate plots in each field and from machine harvest. The data dictionary and data tabs are preceded by an introduction tab that explains the other tab names and contents, team members, pertinent references, and conventions. Data are intermittent, meaning that if measurements were not taken on a date, then there is no date or data entry in the file.

Resources in this dataset:

- Resource Title: 1989 Bushland, TX, east maize growth and yield data. File Name: 1989_East_Maize_Growth_and_Yield(ADC).xlsx.
- Resource Title: 1990 Bushland, TX, east maize growth and yield data. File Name: 1990_East_Maize_Growth_and_Yield(ADC).xlsx.
- Resource Title: 1994 Bushland, TX, east maize growth and yield data. File Name: 1994_East_Maize_Growth_and_Yield(ADC).xlsx.
- Resource Title: 1994 Bushland, TX, west maize growth and yield data. File Name: 1994_West_Maize_Growth_and_Yield(ADC).xlsx.
- Resource Title: 2013 Bushland, TX, west maize growth and yield data. File Name: 2013_West_Maize_Growth_and_Yield(ADC).xlsx.
- Resource Title: 2013 Bushland, TX, east maize growth and yield data. File Name: 2013_East_Maize_Growth_and_Yield(ADC).xlsx.
- Resource Title: 2016 Bushland, TX, east maize growth and yield data. File Name: 2016_East_Maize_Growth_and_Yield(ADC).xlsx.
- Resource Title: 2016 Bushland, TX, west maize growth and yield data. File Name: 2016_West_Maize_Growth_and_Yield(ADC).xlsx.
- Resource Title: 2018 Bushland, TX, west maize growth and yield data. File Name: 2018_West_Maize_Growth_and_Yield(ADC).xlsx.
- Resource Title: 2018 Bushland, TX, east maize growth and yield data. File Name: 2018_East_Maize_Growth_and_Yield(ADC).xlsx.

Weighing Lysimeter Data for The Bushland, Texas Maize for Grain Datasets (10.15482/USDA.ADC/1526333)^21^.

Each lysimeter was equipped with a suite of instruments to sense lysimeter mass, wind speed, air temperature and relative humidity, components of the radiation balance (e.g., net radiation, incoming and reflected shortwave, photosynthetically active radiation (PAR) in some years, incoming and upwelling longwave, thermal infrared emitted by the plant/soil surface), soil heat flux, soil temperature, and soil volumetric water content at certain depths (in later years). This dataset contains lysimeter soil water storage and drainage data, which are converted to equivalent depth of water per unit area from lysimeter mass data, as well as data from in-soil and above-soil sensors. Although a quality control process was used, the evapotranspiration (ET) data in this dataset are considered raw data. Advanced algorithms for detection of precipitation, dew and frost were applied in a separate process to determine higher quality ET values that are reported in files in a dataset entitled “Evapotranspiration and Water Balance Data for The Bushland, Texas Maize for Grain Datasets” (see following section). Those files have “water-balance” in their names. Not all properties were always sensed in any one year; and instruments used changed from season to season, which are reasons that subsidiary datasets and data dictionaries for each season are required. Data are on 5-minute, 15-minute, or daily basis for all days of the year with missing data indicated by #N/A.

There are up to two files for each year, one for east (NE and SE) fields if maize was grown there, and one for west (NW and SW) fields if maize was grown there. Data are in Excel files consisting of an introductory tab followed by data dictionary and data tabs. The data tab for 5-minute data contains only data that were reported on a 5-minute mean basis, typically lysimeter relative water storage, standard deviation of same, rain gauge data, and in the case of subsurface drip irrigation in 2014 and later, the irrigation storage tank water storage, again in mm equivalent depth of water. In the 5-minute data tab, there are columns for data flags for water storage values for each lysimeter, and the meaning of the flags is given in the introductory tab. These flags typically indicate the occurrences of precipitation, dew or frost accumulation, lysimeter counterweight adjustment, lysimeter maintenance, drainage tank emptying, and other occurrences that would have influenced the lysimeter mass other than the evapotranspiration process itself. A tab for 15-minute mean data values contains data for lysimeter relative water storage and all other data from sensors in and above the lysimeter. A daily tab contains daily mean or total data for the same sensors and the corresponding data dictionary explains the units of measure, instruments used, and methods used. The daily data tab is followed by several tabs that are used for data visualization and do not contain data.

Resources in this dataset:

- Resource Title: 1989 Bushland, TX, East Maize Weighing Lysimeter and Microclimate Data. File Name: 1989_East_Maize_Lys_ClimDat.xlsx.
- Resource Title: 1990 Bushland, TX, East Maize Weighing Lysimeter and Microclimate Data. File Name: 1990_East_Maize_Lys_ClimDat.xlsx.
- Resource Title: 1994 Bushland, TX, East Maize Weighing Lysimeter and Microclimate Data. File Name: 1994_East_Maize_Lys_ClimDat.xlsx.
- Resource Title: 1994 Bushland, TX, West Maize Weighing Lysimeter and Microclimate Data. File Name: 1994_West_Maize_Lys_ClimDat.xlsx.
- Resource Title: 2013 Bushland, TX, East Maize Weighing Lysimeter and Microclimate Data. File Name: 2013_East_Maize_Lys_ClimDat.xlsx.
- Resource Title: 2013 Bushland, TX, West Maize Weighing Lysimeter and Microclimate Data. File Name: 2013_West_Maize_Lys_ClimDat.xlsx.
- Resource Title: 2016 Bushland, TX, East Maize Weighing Lysimeter and Microclimate Data. File Name: 2016_East_Maize_Lys_ClimDat.xlsx.
- Resource Title: 2016 Bushland, TX, West Maize Weighing Lysimeter and Microclimate Data. File Name: 2016_West_Maize_Lys_ClimDat.xlsx.
- Resource Title: 2018 Bushland, TX, East Maize Weighing Lysimeter and Microclimate Data. File Name: 2018_East_Maize_Lys_ClimDat.xlsx.
- Resource Title: 2018 Bushland, TX, West Maize Weighing Lysimeter and Microclimate Data. File Name: 2018_West_Maize_Lys_ClimDat.xlsx.

Evapotranspiration, Irrigation, Dew/frost - Water Balance Data for The Bushland, Texas Maize for Grain Datasets (10.15482/USDA.ADC/1526334)^22^.

The water balance data consist of 15-minute and daily amounts of evapotranspiration (ET), dew/frost accumulation, precipitation (rain/snow), irrigation, scale counterweight adjustment, and emptying of drainage tanks, all in mm equivalent depth of water. There are separate data tabs for the daily and 15-minute data, and a third data tab that gives irrigation amounts and irrigation application method for each date on which irrigation was applied. A data dictionary for each data tab explains units of measurement, methods of measurement, methods of irrigation, or other information pertinent to each data column header. The values are the result of a rigorous quality control process involving algorithms for detecting dew/frost accumulations, and precipitation (rain and snow)^15,19^. Changes in lysimeter mass due to emptying of drainage tanks, counterweight adjustment, maintenance activity, and harvest are accounted for such that ET values are minimally affected. The ET, precipitation, and irrigation data in this dataset should be considered to be the best values offered in these datasets. Even though ET data are also presented in the “lysimeter” datasets, the values herein are the result of a more rigorous quality control process. Dew and frost accumulation varies from year to year and seasonally within a year, and it is affected by lysimeter surface condition [bare soil, tillage condition, residue amount and orientation (flat or standing), etc.]. Particularly during winter and depending on humidity and cloud cover, dew and frost accumulation sometimes accounts for an appreciable percentage of total daily ET. An introductory tab explains the other tabs, gives lists of authors and references, and explains conventions and symbols used. It also lists instruments and data recorders used, and longitude and latitude of lysimeter assets, weather station, and field corners. There are up to two files for each year, one for east (NE and SE) fields if maize was grown there, and one for west (NW and SW) fields if maize was grown there.

Resources in this dataset:

- Resource Title: 1989 Bushland, TX. East Maize Evapotranspiration, Irrigation, and Water Balance Data. File Name: 1989_Maize_water_balance.xlsx
- Resource Title: 1990 Bushland, TX. East Maize Evapotranspiration, Irrigation, and Water Balance Data. File Name: 1990_Maize_water_balance.xlsx
- Resource Title: 1994 Bushland, TX. East Maize Evapotranspiration, Irrigation, and Water Balance Data. File Name: 1994_Maize_water_balance.xlsx
- Resource Title: 2013 Bushland, TX. East Maize Evapotranspiration, Irrigation, and Water Balance Data. File Name: 2013_Maize_water_balance.xlsx
- Resource Title: 2016 Bushland, TX. East Maize Evapotranspiration, Irrigation, and Water Balance Data. File Name: 2016_Maize_water_balance.xlsx
- Resource Title: 2018 Bushland, TX. East Maize Evapotranspiration, Irrigation, and Water Balance Data. File Name: 2018_Maize_water_balance.xlsx

Standard Quality Controlled Research Weather Data – USDA-ARS, Bushland, Texas (10.15482/USDA.ADC/1526433)^11^.

This dataset contains 15-minute mean or total quality-controlled^12^ weather data from the USDA-ARS Conservation and Production Laboratory (CPRL), Soil and Water Management Research Unit (SWMRU) research weather station, Bushland, Texas (Lat. 35.186714°, Long. −102.094189°, elevation 1170 m above MSL) for all days in each year. The data are from sensors placed at 2-m height over a level, grass surface mowed to not exceed 0.12-m height and irrigated and fertilized to maintain reference conditions^25,26^. Irrigation was by surface flood in 1989 through 1994, and by subsurface drip irrigation after 1994. Sensors were replicated and intercompared between replicates and with data from nearby weather stations, which were sometimes used for gap filling. Data from a duplicate sensor were used to fill gaps in data from the primary sensor using appropriate regression relationships. Gap filling was also accomplished using sensors deployed at one of the four large weighing lysimeters immediately west of the weather station, or using sensors at other nearby stations when reliable regression relationships could be developed. An important secondary station is the CPRL National Weather Service station. The primary paper^12^ describes details of the sensors used and methods of testing, calibration, inter-comparison, and use. The weather data include air temperature (°C) and relative humidity (%), wind speed (m s^−1^), solar irradiance (W m^−2^), barometric pressure (kPa), and precipitation (rain and snow in mm). Because the large (3 m by 3 m surface area) weighing lysimeters are better rain gages than are tipping bucket gages, the 15-minute precipitation data are derived for each lysimeter from changes in lysimeter mass.

Data are in Excel files, one for each year. An introductory tab explains what are in the other tabs, lists authors and references, explains conventions and symbols, lists instrumentation used, including for the CPRL National Weather Service station, and gives the latitude and longitude of stations. The “15-minute weather” tab gives solar irradiance, air temperature and humidity, wind speed, air pressure, the precipitation at each of the four lysimeters and a mean precipitation value. The “daily precip. data” tab gives precipitation from each lysimeter and the mean precipitation for every day of the year. A data dictionary precedes each data tab. Additional tabs are included for data visualization, including a graphical representation of missing data, and a graphical representation of the weather data in 5-day segments as chosen by the user.

Resources in this dataset (only files for the years in which maize was grown are listed):

- Resource Title: 1989 Bushland, TX, standard 15-minute weather data. File Name: 1989_15-min_weather_SWMRU_CPRL.xlsx.
- Resource Title: 1990 Bushland, TX, standard 15-minute weather data. File Name: 1990_15-min_weather_SWMRU_CPRL.xlsx.
- Resource Title: 1994 Bushland, TX, standard 15-minute weather data. File Name: 1994_15-min_weather_SWMRU_CPRL.xlsx.
- Resource Title: 2013 Bushland, TX, standard 15-minute weather data. File Name: 2013_15-min_weather_SWMRU_CPRL.xlsx.
- Resource Title: 2016 Bushland, TX, standard 15-minute weather data. File Name: 2016_15-min_weather_SWMRU_CPRL.xlsx.
- Resource Title: 2018 Bushland, TX, standard 15-minute weather data. File Name: 2018_15-min_weather_SWMRU_CPRL.xlsx.

Soil Water Content Data for The Bushland, Texas Large Weighing Lysimeter Experiments (10.15482/USDA.ADC/1526332)^10^.

This dataset contains soil water content data developed from neutron probe readings taken in access tubes in each of the four large, precision weighing lysimeters and in the fields surrounding each lysimeter beginning in 1989. Readings were taken periodically with a field-calibrated^13,27,28^ neutron probe at depths from 0.10 m to 2.30 m (maximum of 1.90 m depth in the lysimeters) in 0.20-m depth increments. Periods between readings were typically one to two weeks, sometimes longer according to experimental design, need for data, and weather that could prevent entry into the field. Field calibrations in the Pullman soil series were done every few years. Calibrations typically produced a regression equation with RMSE < = 0.01 m^3^ m^3 ^^29^. Data were used to guide irrigation scheduling to achieve full or deficit irrigation as required by the experimental design. Data may be used to calculate the soil profile water content in mm of water from the surface to the maximum depth of reading. Profile water content differences between reading times in the same access tube are considered the change in soil water storage (∆*S*, mm) during the period in question and may be used to compute evapotranspiration (ET) with accuracy comparable to that for the weighing lysimeter for periods of a week or longer^30^. The soil water balance equation is: ${\rm{ET}}=\triangle S+P+I+F+{\rm{R}}$, where *P* is precipitation during the period, *I* is irrigation during the period, *F* is soil water flux out of the bottom of the soil profile during the period (drainage is a negative value), and *R* is the sum of runon and runoff during the period. Typically, *R* is taken as zero because the fields were furrow diked to prevent runon and runoff during most of each growing season.

Data are in Excel spreadsheets with several tabs, not all of which are data tabs, but each data tab is preceded by a data dictionary tab explaining the measurement units and method for each column of data. The first tab is an introductory tab explaining the other tabs, listing the authors, and key references and listing conventions and explaining symbols. The first data tab gives profile water contents in the top 1.5 m of soil and also in the profile to 2.4-m depth. Profile water contents are given as mean values for each lysimeter (mean of readings in two access tubes) and field (from four to eight access tubes depending on the year, more for later years). Profile water contents are also given as mean values for every two access tubes in the field. There are two files for each year, one for east (NE and SE) fields, and one for west (NW and SW) fields. Depthwise soil volumetric water content (VWC) values are given in a tab for each day of reading, each depth, and each access tube number. The profile water content data are synthesized from these depthwise data. A “Tube Map” tab gives the location for each access tube by its number. Locations are relative to the fields and lysimeters and in later years are given in latitude and longitude. A “Graph” tab allows visualization of the data with one graph for each access tube illustrating the data for all dates of measurement.

Resources in this dataset (only files for the years in which maize was grown are listed):

- Resource Title: 1989 Bushland, TX, east maize volumetric soil water content data. File Name: 1989_East_Maize_Soil-water.xlsx
- Resource Title: 1990 Bushland, TX, east maize volumetric soil water content data. File Name: 1990_East_Maize_Soil-water.xlsx
- Resource Title: 1994 Bushland, TX, east maize volumetric soil water content data. File Name: 1994_East_Maize_Soil-water.xlsx
- Resource Title: 1994 Bushland, TX, west maize volumetric soil water content data. File Name: 1994_West_Maize_Soil-water.xlsx
- Resource Title: 2013 Bushland, TX, east maize volumetric soil water content data. File Name: 2013_East_Maize_Soil-water.xlsx
- Resource Title: 2013 Bushland, TX, west maize volumetric soil water content data. File Name: 2013_West_Maize_Soil-water.xlsx
- Resource Title: 2016 Bushland, TX, east maize volumetric soil water content data. File Name: 2016_East_Maize_Soil-water.xlsx
- Resource Title: 2016 Bushland, TX, west maize volumetric soil water content data. File Name: 2016_West_Maize_Soil-water.xlsx
- Resource Title: 2018 Bushland, TX, east maize volumetric soil water content data. File Name: 2018_East_Maize_Soil-water.xlsx
- Resource Title: 2018 Bushland, TX, west maize volumetric soil water content data. File Name: 2018_West_Maize_Soil-water.xlsx

## Technical Validation

**Fig. 2:**
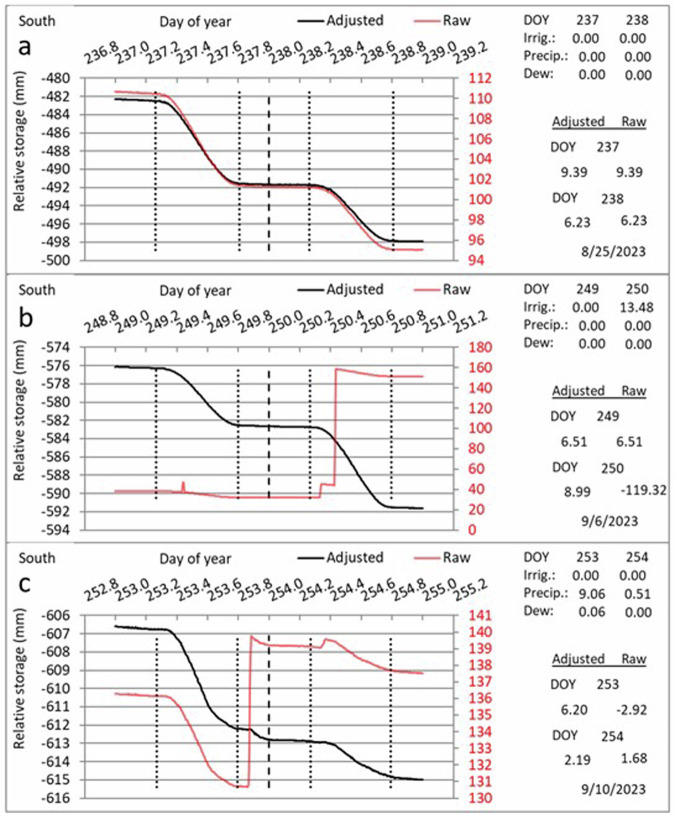
Illustrations of raw lysimeter data (red) showing the change of relative storage (mass) over time due to various events, and corrected data (black) after the events are taken into account such that relative storage change reflects only evapotranspiration. Vertical dotted lines indicate sunrise and sunset; vertical dashed line indicates midnight. (**a**) Change in relative storage over time on two days of year (DOY) when no events other than evapotranspiration caused changes in lysimeter mass. (**b**) Illustration of raw lysimeter data with a spike due to a neutron probe reading on the lysimeter on DOY 249, an irrigation in the morning of DOY 250, and a counterweight adjustment later that morning, and corrected data after these events were taken into account. (**c**) Change in relative storage over time when a rainfall occurred after sundown on DOY 253, and a small rainfall occurred after sunrise on DOY 254, and corrected data reflecting only evapotranspiration.

Numerous studies have been conducted to improve and maintain quality of the Bushland weighing lysimeter data. Weighing lysimeter calibrations have demonstrated accuracy ranging from 0.01- to 0.05-mm equivalent water depth for the lysimeter scales^31,32^. Methods of neutron probe use and calibration for determination of soil water content developed at Bushland have been widely reported in journal articles^13,27,33^ and book chapters^28,34–37^. Studies demonstrated the use of the neutron probe to accurately determine the profile water content from the soil surface to various depths and the equivalency of ET calculated from neutron probe based soil water balance to weighing lysimeter ET for periods of a week or longer^30,31^. Because weighing lysimeters represent a relatively small surface area compared to the much larger field, there is a question of how representative weighing lysimeter ET is of field ET. A network of neutron probe access tubes in the field around a lysimeter was shown to be useful in demonstrating that the lysimeter ET was representative of the field ET when crop cover on the lysimeter was essentially the same as that in the field^31^. Changes in lysimeter apparent relative water storage occurred due to irrigation, precipitation, dew or frost accumulation, scale counterweight adjustments, work on the lysimeter such as neutron probe readings, and other events other than evapotranspiration (ET). These non-ET apparent storage changes must be corrected so that ET can be calculated from the corrected lysimeter storage. Methods for detecting these events and making the corrections were developed and documented, and some are illustrated in Fig. 2^15,19,20^.

The example spreadsheet^20^ available on the Ag Data Commons contains a year-long sequence of 5-minute lysimeter storage data and demonstrates the algorithms and calculations made to correct all the events illustrated in Fig. 2 and others discussed in the 10 papers cited in the spreadsheet posting. Several lysimeter data processing schemes have been published that use steps in addition to or in lieu of those described herein. All still included some form of event flagging and quality control steps. One scheme consisted of five steps: a manual filter (e.g., removal of I, P, or maintenance events), threshold filter (e.g., removal of spikes), median filter (single outliers or smaller spikes), smoothing (moving average with 15-min window), and oscillation threshold filter (further smoothing), where interpolation was used to fill gaps following the manual filter and threshold filter steps^38^. Another scheme^39^ used manual filtering followed by Savitsky-Golay^40^ smoothing of lysimeter storage and its first derivative, which is ET. Some schemes described for non-irrigated bare soil or natural vegetated surfaces assumed that P and ET do not occur simultaneously, so that gap filling of ET was not required during P events^41–44^. We consider our methods to be superior because they do not lose detailed information through application of smoothing. However, our methods may be more time consuming because they require user intervention to apply flags and correct automatically assigned flags where necessary.

Quality assurance and control were also achieved through calibration of relative humidity sensors (using salt solutions and a calibration chamber)^24,37^, heat flux plates^45^, radiation sensors (routinely sent to manufacturers for calibration), anemometers (in a test stand), and tipping-bucket rain gauges (comparison to lysimeter mass change and to U.S. Weather Bureau standard rain gauges). Solar irradiance is compared to theoretical clear-sky radiation both to gauge sensor performance and to check on data time stamping^12,32^. Multiple redundant measurements were made of important properties (air temperature and RH, wind speed and solar irradiance) for intercomparison and replacement of faulty or missing data. Electromagnetic soil water sensors used in 2013 and later years were calibrated for soil water content, temperature, and bulk electrical conductivity^46^, and shown to be comparable in accuracy to the neutron probe^47^.

Plant growth and yield sampling and observation involved replicate (n = 3 or more) plots randomly assigned in each field. Individual values were reported so that means and standard deviations could be computed and statistical analysis applied. Plant growth stages were observed on the replicate plots where V represents vegetative stage, R represents reproductive stage, BL represents Black Layer, and the numbers indicate intermediate stages of V and R^48^. Harvest index was calculated from grain yield and above ground biomass values, in part as a check against values reported in the literature to assess systematic errors^4^.

## Usage Notes

These data may be used to test and calibrate models of maize growth, energy and water balance, water use (ET), and yield, and may be used to develop crop coefficients for use with a reference evapotranspiration model to estimate crop water use^49–54^. Care was taken to ensure that lysimeter ET data were representative of the 4.4 ha fields within which each lysimeter was centered. Therefore, satellite data with 100-m or smaller pixels may be suitable for use with the lysimeter data in testing and calibration of models based on satellite data, and they have been so used^8,9^. That said, the data pertain to the specific location, soil, climate, cultivar, and agronomic practices described in the data sets. Extrapolation to other climates, soils, cultivars, and practices should be done with care. Individual fields were square and somewhat larger than 210 m in width and length, so care should be used when combining satellite data with these data if satellite image pixels are large. Observations of air temperature and relative humidity, wind speed, and solar irradiance taken at the lysimeters should not be used as weather input for simulation models; rather, weather data observed under standard conditions at the research weather station should be used as input to simulation models.

## Data Availability

A custom Excel spreadsheet was used to identify changes in apparent lysimeter water storage caused by other than evapotranspiration (ET) and to correct the lysimeter water storage so that it reflected only ET, enabling calculation of ET (see Fig. 2). The spreadsheet was essentially an embodiment of Eq. 1. The spreadsheet allowed separate tabulation of amounts of precipitation, irrigation, dew and frost accumulation, and drainage tank emptying. The spreadsheet is available on the USDA ARS NAL Ag Data Commons (10.15482/USDA.ADC/26898151)^20^.
